# Editorial: The versatile kisspeptin: advances in cancer, metabolism, and reproduction

**DOI:** 10.3389/fendo.2023.1239694

**Published:** 2023-07-13

**Authors:** Ali Abbara, Moshmi Bhattacharya

**Affiliations:** ^1^ Division of Diabetes and Endocrinology, Section of Investigative Medicine, Imperial College London, London, United Kingdom; ^2^ Department of Medicine, Robert Wood Johnson Medical School, Rutgers University, New Brunswick, NJ, United States

**Keywords:** kisspeptin, reproduction, ovulation, sex, mood, KNDy neurons, IVF

Kisspeptin is increasingly recognized to have several roles in the body including in cancer, metabolism, and reproduction, highlighting it as a versatile peptide. Kisspeptins are peptides encoded for by the *KISS1* gene and signal *via* the G-protein coupled kisspeptin receptor (*KISS1R*). Kisspeptin was first discovered as a regulator of cancer metastasis and then later as a potent regulator of hypothalamic gonadotrophin-releasing hormone (GnRH) activity in the neuroendocrine-reproductive axis. Since then, further roles in the regulation of human sexual behavior, the pathogenesis of metabolic-associated fatty liver disease (MAFLD), and local reproductive functions in the male and female gonads have been recognized. This Research Topic provides an update on kisspeptin and its emerging roles in regulating reproduction and the clinical applications of these findings.

Kisspeptin plays a major role in reproduction as a key regulator of hypothalamic GnRH function. There are two distinct populations of kisspeptin neurons with dedicated functions. Kisspeptin neurons in the arcuate nucleus in animals (analogous to the infundibular nucleus in humans), co-express neurokinin B and dynorphin, and play a major role in regulating pulsatile secretion of GnRH. Kisspeptin neurons in the rostral periventricular area of the third ventricle (RP3V), which includes the anteroventral periventricular (AVPV) nucleus and preoptic area, play a major role in mediating positive feedback from oestradiol to induce the mid-cycle luteinising hormone (LH) surge responsible for instigating ovulation.

Masumi et al. review the role of hypothalamic and peripheral kisspeptin in regulating ovulation, focusing on follicle development, oocyte fertilization and maturation. Stevenson et al. review how oestradiol feedback on kisspeptin neurons is divergent in the two hypothalamic regions during the physiological menstrual cycle to result in follicular development and the mid-cycle LH surge/ovulation. This article also discusses reproductive diseases that could result from dysregulation of kisspeptin neuronal activity. Sharma et al. review available clinical data and future potential of the use of kisspeptin as a trigger of oocyte maturation during *in vitro* fertilization treatment. Finally, Mills et al. explore the emerging role of kisspeptin in regulating sexual behavior and its potential for use in the treatment of hypoactive sexual desire disorder (HSDD). We hope that this Research Topic provides a useful update on kisspeptin in this fast-moving field and helps to stimulate further research in the area.

## Author contributions

All authors listed have made a substantial, direct, and intellectual contribution to the work and approved it for publication.

